# The prevalence of brain abnormalities in boys with central precocious puberty may be overestimated

**DOI:** 10.1371/journal.pone.0195209

**Published:** 2018-04-03

**Authors:** Jong Seo Yoon, Cheol Hwan So, Hae Sang Lee, Jung Sub Lim, Jin Soon Hwang

**Affiliations:** 1 Department of Pediatrics, Ajou University School of Medicine, Ajou University Hospital, Suwon, Korea; 2 Department of Pediatrics, Wonkwang University School of Medicine, Wonkwang University Hospital, Jeonbuk, Korea; 3 Department of Pediatrics, Korea Cancer Center Hospital, Seoul, Korea; Instituto Cajal-CSIC, SPAIN

## Abstract

Brain magnetic resonance imaging (MRI) is routinely performed to identify brain lesions in boys with central precocious puberty (CPP). We investigated the prevalence of CPP in Korean boys and the necessity for routine brain MRI examinations. This retrospective cross-sectional study was conducted from April 2003 to December 2016 at a Korean university hospital. Among 151 boys who were diagnosed with CPP, the data of 138 boys who underwent sellar MRI were evaluated. The mean age of the study subjects was 9.51 ± 0.56 years (<8 years [n = 4] and ≥8 years [n = 134]). We excluded patients who had been previously diagnosed with brain tumors and those who did not undergo a sellar MRI because of refusal or the decision of the pediatric endocrinologist. The main outcome measure was the prevalence of intracranial lesions among boys with CPP. Normal sellar MRI findings were observed in 128 of the 138 boys (93%). Mild brain abnormalities were found in 10 boys (7%), while none of the patients had pathological brain lesions. The prevalence (7%) of intracranial lesions among boys who were healthy, did not have neurological symptoms, and were diagnosed with CPP was different from that previously reported. None of the identified lesions necessitated treatment. Although this was a single country study, we found that the previously reported prevalence of brain lesions in boys with CPP is much higher than the prevalence observed in Korea. This study suggests the need to globally reevaluate the prevalence of pathological brain lesions among male pediatric patients with CPP.

## Introduction

Central precocious puberty (CPP) is the onset of secondary sexual characteristics before the ages of 8 years in girls and 9 years in boys; it is caused by the early activation of the hypothalamic-pituitary-gonadal axis [1]. CPP may be a sign of an existing central nervous system pathology.

The North American and European Pediatric Endocrinology Societies concluded that girls aged <6 years with CPP should undergo brain magnetic resonance imaging (MRI). However, considering that only 2–7% of girls who show an onset of CPP between the ages of 6 and 8 years have unsuspected pathology and only <1% have tumors, it is controversial whether all girls who develop CPP between the ages of 6 and 8 years should undergo brain MRI [2]. In contrast to girls, in whom 90% of CPP is idiopathic, approximately 40–75% of boys with CPP have pathological brain lesions [1, 2]. Thus, brain MRI of all boys with CPP, regardless of their age, is an essential screening tool to exclude pathological brain lesions. The criteria for brain MRI examinations are strongly influenced by the diagnostic criteria of CPP, such as the age at diagnosis. The mean age of pubertal onset has declined over the last two decades globally [3–5]. Even in healthy children, the age at the onset of puberty is lower than it has ever been. However, there has been no change in the diagnostic criteria for CPP, and the prevalence of brain lesions in boys with CPP has remained steady.

Therefore, the purpose of this study was to investigate the prevalence of brain lesions in boys with CPP over the past decade in Korea and to compare it to previously reported data.

## Subjects and methods

### Study participants

The data of 151 boys who were diagnosed with CPP at the Pediatric Endocrine Unit of the Ajou University Hospital (Gyeonggi-do, Korea) between April 2003 and December 2016 were retrospectively analyzed. The inclusion criteria were a diagnosis of CPP before the age of 10 years and the completion of a sellar MRI. The exclusion criteria included neurofibromatosis, congenital adrenal hyperplasia associated with precocious puberty, a previously identified brain tumor (1 patient with astrocytoma, aged 1 month, and 1 patient with optic glioma, aged 15 months), hydrocephalus, and trauma (n = 8). Moreover, we excluded five patients who refused to undergo an MRI. A total of 138 boys were included in the final analysis.

**Fig 1:**
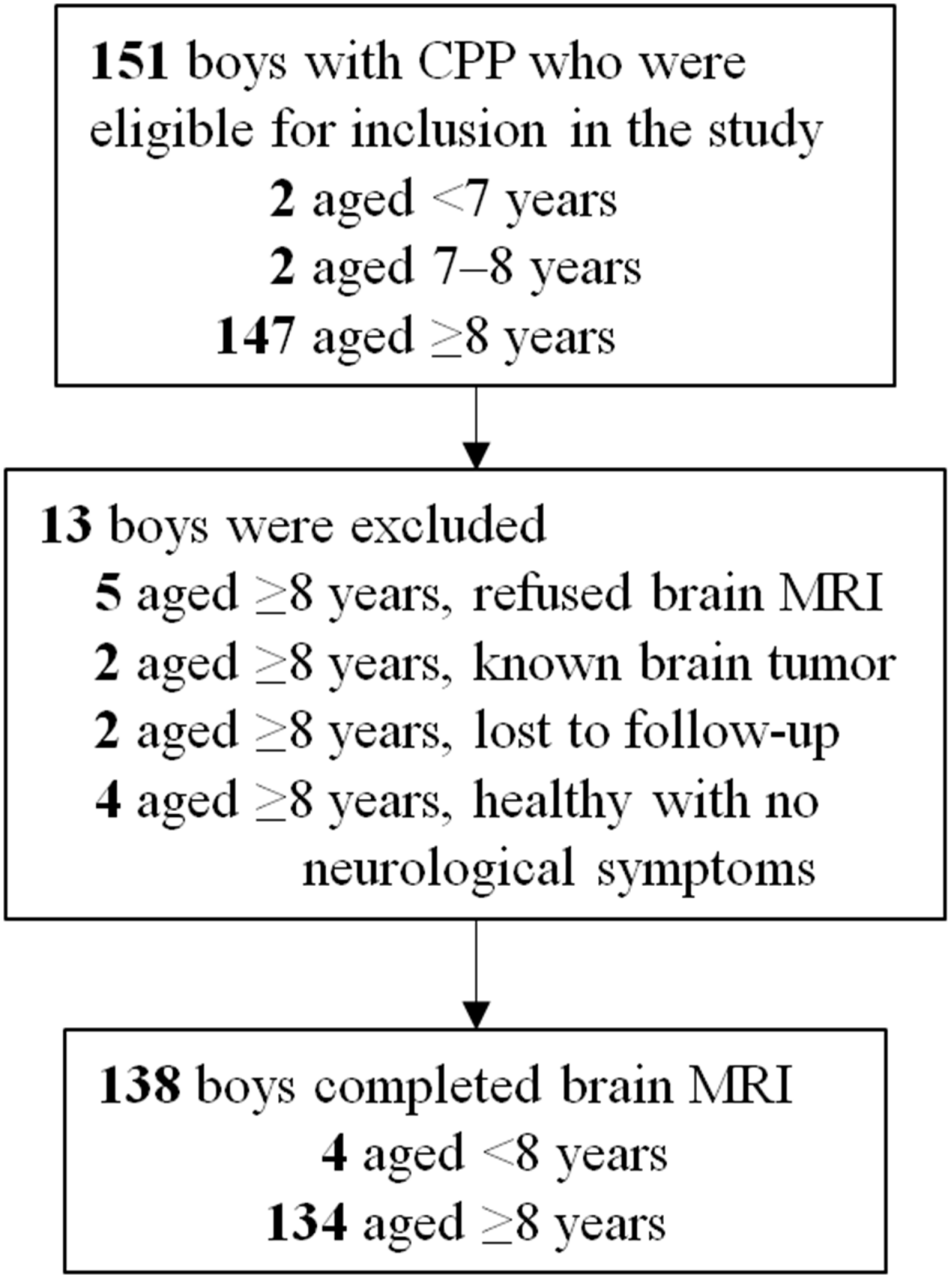
Study flow chart. CPP: central precocious puberty, MRI: magnetic resonance imaging.

The patients were divided into 2 age groups: <8 years (n = 4), and ≥8 years (n = 134; Fig 1).

The study protocol was approved by the institutional review board of the Ajou University Hospital (AJIRB-MED-MDB-16-459). The requirement for informed consent was waived owing to the retrospective nature of this study. This research was conducted in accordance with the principles of the Declaration of Helsinki.

### Diagnosis of central precocious puberty

The CPP diagnosis was based on a clinical evaluation that included a full clinical history of the patients and caregivers, followed by Tanner staging conducted by a pediatric endocrinologist. It was confirmed on the basis of an increase in the testicular volume before the age of 9 years, a bone age advancement >2 standard deviations above the chronological age, and a peak luteinizing hormone (LH) level of ≥5 mIU/mL after a gonadotropin-releasing hormone (GnRH) stimulation test [6].

Height was measured to the nearest 0.1 cm using a Harpenden stadiometer (Holtain Ltd., Crymych, Wales, UK), and weight was measured to the nearest 0.1 kg using a digital scale. The body mass index (BMI) was calculated by dividing the weight by the square of the height (kg/m^2^). The standard deviation scores (SDS) for height, weight, and BMI were calculated based on the 2007 Korean National Growth Charts [7]. Bone age was assessed via radiography of the left hand, according to the method established by Greulich and Pyle [8]. Serum LH and follicle-stimulating hormone (FSH) levels were measured using an immunoradiometric assay (BioSource, Nivlles, Belgium) with detection limits of 0.1 IU/L and 0.2 IU/L, respectively. Testosterone levels were measured using a radioimmunoassay with a detection limit of 0.01 ng/mL. The GnRH stimulation test was performed during the day, and serum LH and FSH levels were determined at 0 min, 30 min, 45 min, 60 min, and 90 min after the GnRH injection (100 μg Relefact; Sanofi-Aventis, Frankfurt, Germany).

All boys diagnosed with CPP underwent sellar MRI, using before and after gadolinium-enhanced T1- and T2-weighted images in axial, coronal, and sagittal sections. The sellar MRI examination was performed using two kinds of machines: prior to 2016: GE scanner, 1.5 (HDxt) and 3.0 Tesla magnet (Discovery MR750w), GE healthcare, the United States; Phillips Achieva, 3.0 Tesla magnet, Philips Medical Systems, Israel; and after 2016: GE scanner, 3.0 Tesla magnet (Discovery MR750w), GE healthcare, the United States; Phillips Achieva, 3.0 Tesla magnet, Philips Medical Systems, Israel.

### Statistical analyses

Statistical analyses were performed using SPSS version 21.0 (IBM Corp., Armonk, NY, USA). The Mann-Whitney *U* test was used to compare the characteristics between boys with normal sellar MRI findings and those with abnormal findings. Statistical significance was defined as a *P*-value <0.05. The results are reported as means ± standard deviations, unless otherwise stated.

### Data limitations

When diagnosing boys with CPP, it is difficult to determine the exact start time of the increase in testicular volume. Therefore, at the time of CPP diagnosis, the determination of the age at pubertal onset can be a challenge in boys. The CPP diagnosis after the first observation of pubertal symptoms by parents and the first consultation with an endocrinologist is frequently spaced aparty by 1.5 years [9, 10]. The same difficulties were encountered in this study; we included boys with CPP who were diagnosed before the age of 10 years.

## Results

**Fig 2:**
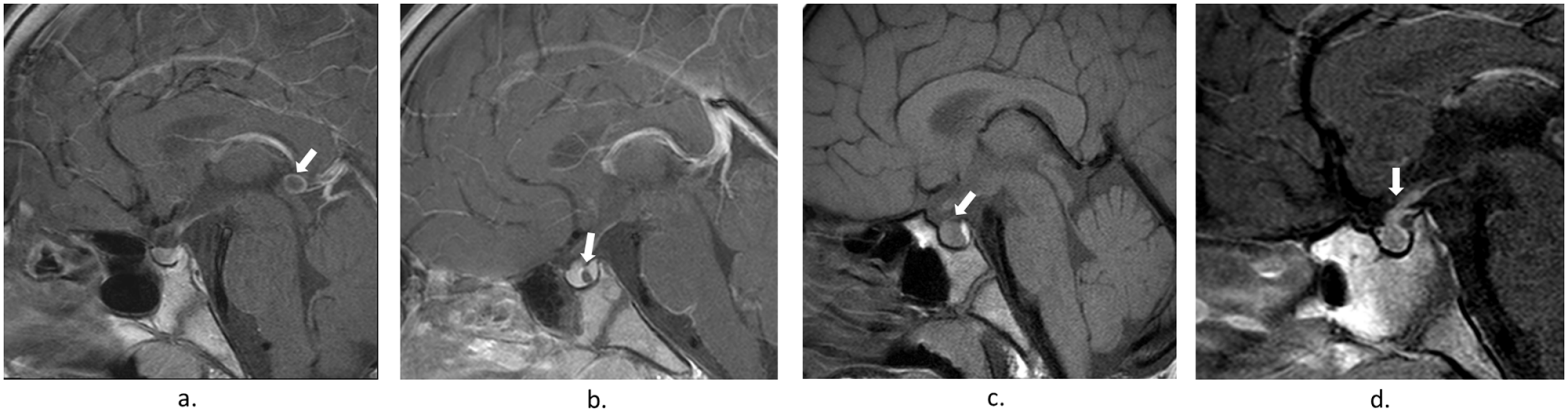
Sellar magnetic resonance imaging of boys with incidental findings. a. Pineal cyst, b. Rathke’s cleft cyst, c. Pituitary hyperplasia, d. Pituitary stalk thickening.

The brain MRI findings of the 138 boys with CPP according to age groups are presented in Table 1. MRI revealed central nervous system abnormalities in 6 boys (4.3%). All MRI findings were considered incidental findings that were not related to early puberty but rather indicated pituitary hyperplasia (n = 3), thickening of the pituitary stalk (n = 1), a Rathke’s cleft cyst (n = 1), and pineal cysts (n = 5; Fig 2). The boys with CPP showing incidental findings underwent ≥2 MRI scans during the follow-up period (mean, 6 months). None of the boys showed central nervous system-related symptoms or underwent surgery.

**Table 1:** Patient characteristics according to the age at the onset of puberty.

	Onset of puberty (years)	
	Boys (n = 138)	
	<8 y (n = 4)	≥8 y (n = 134)
**Normal findings (n = 132)**	4	124
Suspected pituitary gland hyperplasia	0	3
Thickening of the pituitary stalk	0	1
**Newly diagnosed findings (n = 6)**		
Rathke’s cleft cyst	0	1
Pineal cyst	0	5

Table 2 depicts the differences in clinical and biochemical characteristics between cases with normal and those with incidental MRI findings. We found no significant differences for the mean age at diagnosis, height (SDS), weight (SDS), BMI (SDS), Tanner stage, bone age (SDS), basal LH levels, basal FSH levels (mIU/mL), and testosterone levels (ng/mL).

**Table 2:** Comparison of clinical and biochemical characteristics according to brain magnetic resonance imaging findings.

	Boys			
	Total (n = 138)	A	B	*P*-value
		(n = 128)	(n = 10)	
**Age at CPP diagnosis (years)**	9.51 ± 0.56	9.52 ± 0.58	9.46 ± 0.29	0.12
**Height (SDS)**	1.14 ± 1.23	1.14 ± 1.22	1.14 ± 1.35	0.98
**Weight (SDS)**	1.12 ± 0.82	1.12 ± 0.82	1.05 ± 0.82	0.73
**BMI (SDS)**	0.87 ± 0.85	0.88 ± 0.85	0.77 ± 0.85	0.80
**Tanner stage**				
**Testicular volume**	5.28 ± 1.78	5.27 ± 1.77	5.30 ± 2.11	0.80
**Pubic hair**	1.30 ± 0.53	1.30 ± 0.52	1.30 ± 0.68	0.76
**Bone age (SDS)**	3.50 ± 1.33	3.53 ± 1.34	3.06 ± 1.14	0.39
**Basal LH (mIU/mL)**	1.00 ± 1.21	1.00 ± 1.24	1.00 ± 0.77	0.55
**Basal FSH (mIU/mL)**	3.24 ± 7.50	3.25 ± 7.78	3.12 ± 1.40	0.12
**Peak LH (mIU/mL)**	16.67 ± 9.36	16.50 ± 9.12	18.70 ± 12.44	0.81
**Testosterone (ng/mL)**	0.66 ± 1.19	0.65 ± 1.21	0.69 ± 0.94	0.57

A: Group with normal findings on brain magnetic resonance imaging. B: Group with new abnormal findings on brain magnetic resonance imaging. BMI: body mass index, CPP: central precocious puberty, FSH: follicle stimulating hormone, LH: luteinizing hormone, SDS: standard deviation score

The temporal distribution of patients diagnosed with both CPP and brain abnormalities are presented in Table 3. The number of boys diagnosed with CPP and the number of incidental findings significantly increased since 2012.

**Table 3:** Annual distribution of patients diagnosed with central precocious puberty and incidental findings on sellar magnetic resonance imaging.

Year	CPP (n)	Incidental findings on sellar MRI (n)	CPP with incidental findings (%)
2016	31	Pineal cyst, 3 Rathke’s cleft cyst, 1 Thickening of the pituitary stalk,1	16.12
2015	15	Pineal cyst, 1	6.67
2014	31	Pineal cyst, 1	3.23
2013	21	Suspected pituitary gland hyperplasia, 2	9.52
2012	10	-	-
2011	8	-	-
2010	4	-	-
2009	5	Suspected pituitary gland hyperplasia, 1	20.00
2008	3	-	-
2007	6	-	-
2006	1	-	-
2004	2	-	-
2003	1	-	-
**Total**	**138**	**10**	**7.25**

CPP: central precious puberty, MRI: magnetic resonance imaging

## Discussion

The known causes of CPP include idiopathic organic brain lesions, hypothalamic hematoma, brain tumors, and hydrocephalus [1, 11–14]. The predictors of organic brain lesions in CPP patients remain unknown; therefore, pediatric endocrinologists routinely recommend brain MRI for all children with CPP. The prevalence of brain lesions is much higher in boys than in girls with CPP [11, 12, 15, 16]; thus, brain MRI is recommended more frequently for boys than girls. We conducted sellar MRI examinations in boys with CPP covering 14 years (2003–2016) and obtained results that are significantly different to those previously reported (showing that approximately 40–75% of boys with CPP have pathological brain lesions) [1, 2]. In the present study, the MRI results did not reveal any lesions that required interventions, and all findings were classified as incidental findings.

A possible association between CPP and Rathke’s cleft cyst has been reported in previous studies [17, 18], although this condition rarely affects children and adolescents; moreover, the incidence of pediatric Rathke’s cleft cyst is unknown [19]. Lim et al. reported that 8 of 44 patients with Rathke’s cleft cyst (18%) also had CPP [17]. However, in this study, we identified a Rathke’s cleft cyst in only 1 of 138 patients with CPP (0.7%). Our case did not exhibit any clinical symptoms and did not require treatment. Pineal cysts are common in the pediatric population, with a higher prevalence among girls and older children [20]. All pineal cysts identified in this study were benign and did not require treatment.

All MRI findings of the present study are also observed in healthy individuals; thus, it remains unclear whether they were causally related to CPP. Pituitary hyperplasia is a frequent cause of incidental findings and may cause precocious puberty; however, the incidence of pituitary hyperplasia in the general population is unknown [21]. In the present study, pituitary hyperplasia was diagnosed via sellar MRI alone and was defined as a bulging contour of the pituitary gland with a height >6 mm [22]. The follow-up period of our study was 52.3 ± 34.70 months (range, 20.0–89.0 months); within this period, no clinical findings of pituitary adenomas were obtained. Therefore, findings indicative of pituitary hyperplasia were considered to suggest normal glands.

We were able to compare our results with the MRI data of girls with CPP who were treated at the same hospital during the same period. During the study period, a total of 455 pediatric patients with CPP (317 girls and 138 boys) underwent sellar MRI at the hospital. The mean age of the girls with CPP was 6.89 ± 1.60 years. Normal sellar imaging findings were observed for 308 of the 317 girls (97.2%). CPP-related incidental findings were found in 9 girls (2.8%). The imaging studies revealed only cases of Rathke’s cleft cysts (n = 7) and pineal cysts (n = 2). Other findings, such as those of suspected pituitary gland hyperplasia (n = 12), suspected pituitary hypoplasia (n = 2), and suspected microadenoma (n = 3), were considered normal findings. In both girls and boys with CPP, suspected pituitary gland hyperplasia, Rathke’s cleft cyst, and pineal cyst were the main incidental findings. However, we were unable to ascertain how these findings are related to CPP.

Our data revealed an increasing trend in the number of boys with CPP and the number of incidental findings since 2012. However, the increasing number of boys with CPP might be related to the increase in the number of sellar MRI examinations during the study period; moreover, since this study was a single institution study, it was difficult to ascertain the annual incidence of incidental findings. According to Kim et al., the annual incidence of CPP in boys in Korea increased from 0.3 to 1.2 per 100,000 boys between 2004 and 2010, although this increase was not significant [5]. In this study, we saw a significant difference in the number of incidental findings before and after 2011. It is likely that the age at the onset of puberty is decreasing in both girls and boys in Korea. Our findings indicate a need for up-to-date nationwide statistics on the annual incidence of CPP in boys in Korea.

This study reported that pathologic brain lesions are rare in Korean boys with CPP. This is in contrast to the previously published prevalence of 40–75% [1, 2]. There are two main potential explanations for the significant difference between the previously published prevalence of brain lesions among boys with CPP and the prevalence obtained in this study. First, the most recent study on CPP prevalence was conducted about two decades ago. Although the reasons remain unknown, the mean age at pubertal onset has been declining, and the prevalence of CPP has been increasing in many countries [4, 5, 23]. It is known that 10% of girls and 40–75% of boys with CPP show brain abnormalities [1, 11]; however, it is unclear how the increase in the prevalence of CPP is linked to the increase in brain abnormalities. Second, the previously reported prevalence of brain lesions in boys with CPP (of 40–75%) was based on a smaller number of subjects when compared to the current study.

The prevalence of CPP in Korean boys has not shown a significantly increasing trend [5]; therefore, we should consider the influence of sociocultural factors besides the underlying causes such as pathophysiologic mechanisms. Generally, it is difficult for parents to identify the beginning of puberty in boys when compared to girls; therefore, cases of CPP could be underrecognized. In Korea, CPP is a social issue, and many parents are keen on addressing this issue. In the present study, a large number of boys were able to visit the hospital with only signs of CPP, and subsequently, the diagnosis was confirmed.

It is important to recognize the relationship between CPP and brain lesions at an early age. As in previous studies, this study also showed that brain lesions manifest at young ages. The children were diagnosed with neurological problems before they were diagnosed with CPP. In the present study, most patients with CPP were around 9 years of age, whereas the onset of puberty was estimated to be around 8 years of age. In this age group, there were no brain abnormalities that required intervention. Moreover, no new brain lesions requiring treatment were detected. This is very different to the previously reported prevalence of brain abnormalities (40–75%) in boys with CPP [1, 2].

The present study has several limitations. First, the prevalence of brain lesions across ages remains unclear because only few subjects aged <7 years were included. Second, this study was conducted in a single country and at a single institution; however, the results are meaningful owing to the large number of subjects and the period of data analysis (14 years). Third, we examined only Korean children, and it is possible that the regional prevalence of brain lesions in CPP cases is related to the study population, ethnicity, healthcare system, and social factors (e.g., access to medical care and resources to diagnose CPP).

## Conclusion

In conclusion, the present study revealed a significantly lower prevalence of brain lesions among boys with CPP than that previously reported. Although it is possible that there are regional or ethnic differences in the prevalence of CPP, we believe that the prevalence of pathological brain lesions in CPP cases has been overestimated. Since this study was only conducted in a Korean population, the results should be reevaluated in other countries. The findings of this study suggest that the routine use of brain MRI to screen all patients with CPP should be reconsidered, in particular in patients who are healthy, neurologically asymptomatic, and boys aged ≥8 years.
